# Single-isocenter multiple-target stereotactic radiosurgery for multiple brain metastases: dosimetric evaluation of two automated treatment planning systems

**DOI:** 10.1186/s13014-022-02086-3

**Published:** 2022-07-01

**Authors:** Giorgio Hamid Raza, Luca Capone, Paolo Tini, Martina Giraffa, Piercarlo Gentile, Giuseppe Minniti

**Affiliations:** 1 Department of Medicine, Surgery and Neurosciences, University of Siena, Siena, Italy; 2 UPMC Hillman Cancer Center, San Pietro Hospital FBF, Via Cassia 600, 00189 Rome, Italy; 3 IRCCS Neuromed, 86077 Pozzilli, IS Italy

**Keywords:** Brain metastases, Stereotactic radiosurgery, Single-isocenter multiple-targets radiosurgery, DCAT, VMAT

## Abstract

**Purpose:**

Automated treatment planning systems are available for linear accelerator (linac)-based single-isocenter multi-target (SIMT) stereotactic radiosurgery (SRS) of brain metastases. In this study, we compared plan quality between Brainlab Elements Multiple Brain Metastases (Elements MBM) software which utilizes dynamic conformal arc therapy (DCAT) and Varian HyperArc (HA) software using a volumetric modulated arc therapy (VMAT) technique.

**Patients and methods:**

Between July 2018 and April 2021, 36 consecutive patients ≥ 18 years old with 367 metastases who received SIMT SRS at UPMC Hillman Cancer San Pietro Hospital, Rome, were retrospectively evaluated. SRS plans were created using the commercial software Elements MBM SRS (Version 1.5 and 2.0). Median cumulative gross tumor volume (GTV) and planning tumor volume (PTV) were 1.33 cm^3^ and 3.42 cm^3^, respectively. All patients were replanned using HA automated software. Extracted dosimetric parameters included mean dose (D_mean_) to the healthy brain, volumes of the healthy brain receiving more than 5, 8,10, and 12 Gy (V_5Gy_, V_8Gy_, V_10Gy_ and V_12Gy_), and doses to hippocampi.

**Results:**

Both techniques resulted in high-quality treatment plans, although Element MBM DCAT plans performed significantly better than HA VMAT plans, especially in cases of more than 10 lesions). Median V_12Gy_ was 13.6 (range, 1.87–45.9) cm^3^ for DCAT plans and 18.5 (2.2–62,3) cm^3^ for VMAT plans (*p* < 0.0001), respectively. Similarly, V_10Gy_, V_8Gy_, V_5Gy_ (*p* < 0.0001) and median dose to the normal brain (*p* = 0.0001) were favorable for DCAT plans.

**Conclusions:**

Both Elements MBM and HA systems were able to generate high-quality plans in patients with up to 25 brain metastases. DCAT plans performed better in terms of normal brain sparing, especially in patients with more than ten lesions and limited total tumor volume.

## Introduction

The clinical management of patients with brain metastases has been changed substantially in the last years, with a shift away from whole brain radiation therapy (WBRT) to stereotactic radiosurgery (SRS). SRS has become the recommended treatment for patients with a limited number of brain metastases, yielding an *equivalent survival* but lower risk of long-term neurocognitive decline as compared with SRS plus WBRT [1, 2]. Similar survival and preservation of neurocognitive function has been demonstrated in patients receiving SRS for more than five metastases [2]. Of note, recent guidelines have suggested that SRS may be used for patients with a higher number of brain metastases (5–10) with a cumulative tumor volume < 15 ml [6].In patients treated with frameless linear accelerator (LINAC)-based SRS, dynamic conformal arc therapy (DCAT) and volumetric modulated arc therapy (VMAT) are usually used for delivering highly conformal radiation doses. One isocenter is typically placed at each lesion which is treated separately; however, single-target approaches require several treatment sessions and long treatment time.

More recently, both VMAT and DCAT techniques have been used for the simultaneous treatment of multiple lesions using a single isocenter. In single-isocenter multiple-target (SIMT) linear accelerator (linac)-based SRS, all arc groups share a single isocenter located at the geometrical center of all lesions and each metastasis is treated by one group of arcs; dose delivery accuracy and conformality are achieved through the use of noncoplanar arcs and simultaneous variation of multileaf collimator (MLC) leaf positions. SIMT SRS, using either DCAT or VMAT techniques provides excellent plan dosimetry and conformity consistent with those achieved with single-target SRS [7–17]. A few clinical studies have shown that SIMT SRS is an effective and safe approach in patients with multiple brain metastases [18–20].

SIMT linac-based SRS approach has been implemented in commercially available software packages using either DCAT or VMAT techniques. Clinically available dedicated systems include Brainlab Elements Multiple Brain Mets SRS treatment planning (Elements MBM, Brainlab, Munich, Germany) which utilizes DCAT technique, and HyperArc (Varian Medical System, Palo Alto, CA, U.S.) and Monaco HD treatment planning systems (Elekta, Stockholm, Sweden) using VMAT technique.

A few studies have shown high plan quality in terms of target coverage and healthy brain sparing for both SIMT DCAT and VMAT SRS systems in patient with up to ten brain metastases; however, plan comparison showed differences of plan quality across the studies [21–25]. Liu et al. [25] showed that SIMT VMAT SRS resulted in better conformity and volume of normal brain receiving 12 Gy (V_12Gy_) in patients with up to ten brain metastases, whereas other studies showed that DCAT plans perform better than VMAT plans in terms of healthy brain sparing and treatment efficiency [21–23].

In the current study, we have compared the efficiency of Elements MBM and HyperArc treatment planning software modules in terms of plan quality metrics, organs at risk (OARs) and healthy brain sparing for patients with multiple brain metastases extending the use of the two systems to patients with up to 25 lesions.

## Patients and methods

Thirty-six consecutive patients ≥ 18 years old with 2–25 brain metastases from various primary cancers who received single-isocenter DCAT SRS between July 2018 and April 2021, at UPMC Hillman Cancer Center San Pietro Hospital, Rome, were retrospectively evaluated. In total, 367 metastases with a major axis diameter < 2 cm were included. Tumor and treatment characteristics are shown in Table 1. With a median number of 9 (2–25) lesions, median cumulative GTV and PTV volume were 1.33 cm^3^ (0.23–4.67) and 3.42 cm^3^ (0.61–9.37), respectively. All patients were treated with frameless linear accelerator (linac)-based single-fraction SRS using a commercial stereotactic mask fixation system (Brainlab, Feldkirchen, Germany). The gross tumor volume (GTV) was contoured on post-contrast thin-slice (0.6–1 mm) gadolinium-enhanced T1-weighted axial magnetic resonance imaging (MRI) sequences fused to the treatment planning computed tomography (CT), which was acquired at 0.625 mm slice spacing. The treatment was performed within 7 days from the MRI. The planning target volume (PTV) was generated by the geometric expansion of GTV plus 1 mm to compensate for uncertainties. Prescribed doses (PD) were 20 Gy for most lesions, maximum doses to the brainstem, optic apparatus, and lens were 12 Gy, 8 Gy, and 2 Gy, respectively. Limiting the volume of healthy brain that received 12 Gy (V_12Gy_) which is a predictor of brain toxicity was used as quality index for optimizing treatment plans. All treatment plans were optimized to deliver the PD at least at 98% of the volume of each PTV, with the covering 2% of each PTV (PTV D_2%_) receiving less than 130% of the PD. After optimization plans with D_2%_ of the cumulative PTV greater than 135% of the PD were not accepted for treatment. All treatments were performed with Varian TrueBeam Novalis Tx (BrainLAB AG, Feldkirchen, Germany and Varian, CA, USA) equipped with HD120 MLC (Varian, CA, USA). The accelerator is equipped with CBCT (Varian CA USA) and Exactrac vs 1.5 (BrainLAB AG).

**Table 1:** Summary of patient charateristics and treatment parameters

Parameter	No
**Number of patients**	36
**Median age**	52
**Sex (F/M)**	19/17
**Histology**	
Lung	15
Breast	9
Melanoma	7
Kidney	4
Sarcoma	1
**No of lesions per patient**	
2–9 lesions	19
10–25 lesions	17
**Tumor location**	
Frontal	27%
Parietal	18%
Temporal	22%
Cerebellar	16%
Occipital	17%
**Radiosurgical dose**	
18 Gy	11
20 Gy	356
**Gross tumor volume (cm**^**3**^**)**	
Median	0.16
Range	0.07–2.1
Median total GTV	1.33
Range	0.23–4.67
**Planning tumor volume (cm**^**3**^**)**	
Median	0.37
Range	0.15–1.18
Median total PTV	3.42
Range	0.61–9.37

### DCAT planning

SRS plans were created using the commercial software Elements MBM SRS (Version 1.5 and 2.0, Brainlab AG) which offers a highly automated planning workflow for single isocenter DCAT treatments of multiple brain metastases. Characteristics of the software have been previously described [31]. In brief, 10 non-coplanar DCAT beams for 5 preset yaw angle couch positions are used by the Elements software after the isocenter location is automatically placed at the center of mass of all target volumes. The start and stop angles of each arc are first set to default values (10° to 170° when couch angle ranges from 0° to 90° and 190° to 350° when couch angle ranges from 270° to 360° (IEC 61217convention) and then automatically modified during optimization changing different beam parameters, including aperture opening, collimator rotation, arc angle and length, and beam weighting in order to attain the prescribed dose for every lesion with the highest conformity possible and to minimize the risk of dose overlap, e.g. in case of two neighboring metastases. Two independent arcs were usually used per couch angle, with the algorithm that automatically determines which targets are going to be treated conformally through each arc. For treatment efficiency, it is attempted to treat as many targets as possible during every arc. In addition, metastases lining up in the direction of leaf motion are automatically not treated simultaneously to restrict normal tissue exposure. Final dose distributions are calculated on a 1 mm grid using a pencil beam algorithm. In addition, two options are available in Elements MBM SRS. The first option permits the correction of the dose inhomogeneity; it has been applied to all plans to avoid variations of PTV D_2%_ larger than 135% of the prescribed dose. The second one allows the addition of extra arc for each lesion when dose boundaries are not respected; however, the “extra arc option” was used rarely, because this option increases the number of MUs while reducing the treatment efficiency.

In our experience the software produces an increase of the maximum dose in the case of two or more lesions quite close themselves, that might be larger than the maximum dose allowed by our treatment policy (PTV D_2%_). For this reason, for PTVs closer than 5 mm, the contours have been merged, so the software consider them as only one PTV. The maximum dose resulted less with a little increase of the dose at the healthy brain.

### Hyperarc VMAT planning

All patients were replanned using Varian HyperArc (HA) (Version 15.6, Varian Medical Systems, Palo Alto, Ca) automated software which utilizes VMAT technique. As for Elements MBM, the isocenter was set to the geometrical average of the centers-of-mass of all target volumes. The set of treatment fields consists of one 360° full arc and up to three 180° half arcs with fixed angles of couch rotation of 0°, 45°, 315° and 270° (Varian IEC scale), while the collimator rotation is optimized during planning to obtain a better plan geometry. Dose distributions were calculated on a 1 mm grid step using the anisotropic analytical algorithm (AAA) implemented in Eclipse TPS (version 15.6Varian Medical Systems, Palo Alto, Ca). With the aim to reduce the dose to the healthy brain, plan optimization can be achieved by Photon Optimizer (PO) with the use two different algorithms, the stereotactic radiosurgery normal tissue objective Auto (SRS NTO) and the automatic lower dose objective (ALDO). SRS NTO algorithm, which generates virtual shells around the target volumes, was used to improve dose falloff and minimize the dose bridging effect between targets. ALDO algorithm is designed to increase PTV dose coverage; however, in the current study, VMAT plans were generated without the use of ALDO algorithm, because it does not allow to limit the upper dose to the targets, e.g. PTV D_2%_ < 130% of prescribed doses, which is the maximum dose allowed in our center. To obtain better plans without ALDO, together with PTV lower constraints that are automatically inserted by software, PTV upper constraints and brain minus PTV constraints have been added. Similarly, OARs (brain stem, hippocampi, optic pathways) upper constraints have been added in case they were closed to PTVs. Finally, during optimization, the priorities of the SRS NTO and constraints have been modified to obtain the better plan allowed by our treatment policy.

### Plan comparison and data analysis

Treatment plans were evaluated by comparing dosimetric indices, dose–volume metrics, and plan efficiency indicators derived from the DVHs for target coverage and sparing of OARs. Dose distribution conformity to the shape and the size of the lesions was assessed by the Paddick conformity index (CI) [26]. The dose fall-off outside the target was assessed by the gradient index (GI), which describe the decrement of the dose in the high-dose region (50% and above).

Indices were defined as follows:

$$CI = \frac{{V_{PI,PTV} }}{{V_{PTV} }}\frac{{V_{PI,PTV} }}{{V_{PI} }}$$

where V_PI_ refers to the volume covered by the 100% of the prescription dose, V_PI,PTV_ is PTV volume covered by the 100% of the prescription dose, and V_PTV_ is the PTV volume. This index represents the degree to which a tumor is covered by a specified isodose curve. A score of 1 corresponds to an ideal isodose conformity to the target volume.

$$GI = \frac{{V_{50\% } }}{{V_{PI} }}$$

where V_50%_ is the volume covered by 50% of the prescription dose, and V_PI_ is the volume covered by 100% of the prescription dose. The GI should be as low as possible. A perfect treatment plan must have a value of the GI around 1.

Based on the significant correlation between the V_12Gy_, defined by brain minus GTV, and the risk of radionecrosis following brain SRS, this dose metric parameter was used as key factor to assess and compare plan quality. For obtaining a better evaluation of the healthy brain V_12Gy_, the rings around the lesions that include the isodose V_12Gy_ have been automatically contoured around each GTV. For overlapping V_12Gy_ due to close lesions, brain volumes were merged to create a cluster. In addition, mean dose (D_mean_) to the healthy brain, volumes of the healthy brain receiving more than 5, 8 and 10 Gy (V_5Gy_, V_8Gy_ and V_10Gy_), and doses to OARs, including hippocampi and optic apparatus, were evaluated. Treatment planning times and estimated delivery times were used to assess treatment efficiency.

A paired 2-tailed Wilcoxon signed-rank test was used to compare the data for the original DCAT vs. simulated VMAT plans for all dosimetric parameters of target coverage and to the OARs. Mean and median statistics are reported for each parameter, but statistics are reported upon the median because the Wilcoxon signed-rank test was used. A value of *p* < 0.05 was considered statistically significant.

## Results

Both Elements MBM and HA software were able to achieve excellent plan dosimetry and conformity. Table 2 summarizes in detail the dosimetric characteristics of DCAT and VMAT plans. Mean target coverage by prescription dose was 98.9% for DCAT plans and 99.1% for VMAT plans. All plans have been optimized with at least 98% of each PTV volume covered by the prescription dose, as required by our treatment policy. In this condition 100% of each GTV volume is covered by the prescription dose for all plans with both software systems.

**Table 2:** Summary of dosimetric parameters of DCAT and VMAT plans

Parameter	DCAT	VMAT	*p*
**Median target coverage**	98.9%	99.1%	NS
Range	95.8%-100%	96.3–100%	
**Paddick conformity index**			
Mean	0.72 (0.09)	0.67 (0.10)	0.005
Range	0.52–0.86	0.50–0.85	
**Gradient index**			
Mean	4.75	5.61	< 0.0001
Range	3.45–7.13	3.64–8.37	
**PTV dose (Gy)**			
Mean (SD)	23.3 (0.23)	24.0 (0.8)	0.0001
Median	23.3	24.0	
Range	22.9–23.9	22.5–25.4	
**PTV D2%**			
Mean (SD)	25.5 (0.4)	26.3 (1.2)	0.001
Median	25.4	26.5	
Range	25.4–27	23.4–28.6	
**Brain**			0.0001
Mean dose (SD)	2.64 (1.3)	3.01 (1.5)	
Median dose (SD)	2.37	2.74	
Range	0.65–5.62	0.55–5.85	
**V12Gy**			< 0.0001
Mean (SD)	14.7 (11.5)	21.8 (17.2)	
Median	13.6	18.5	
Range	1.87–45.9	2.2–62.3	
**V10Gy**			< 0.0001
Mean (SD)	22.3 (17.4)	33.7 (28.9)	
Median	17.4	27.9	
Range	2.6–74.2	2.9–100.6	
**V8Gy**			< 0.0001
Mean (SD)	39.4 (38.1)	59.8 (52.8)	
Median	26.6	46.0	
Range	3.7–157	4.6–184	
**V5Gy**			< 0.0001
Mean (SD)	141.2 (164.6)	211.0 (212.7)	
Median	73.2	142.3	
Range	8.18–701.8	11.3–707.8	
**Right hippocampus**			0.0005
Mean dose (SD)	2.6 (1.7)	3.2 (2.2)	
Median dose	1.9	2.2	
Range	0.6–6.9	0.6–8.6	
**Left hippocampus**			0.0001
Mean dose (SD)	2.7 (1.3)	3.4 (1.9)	
Median dose	2.1	2.7	
Range	0.5–6.4	0.5–9.8	

*DCAT* Dynamic Conformal Arc Therapy, *VMAT* Volumetric Target Volume, *PTV* Planning Target Volume, *PTV D2*% dose received by 2% of PTV, *SD* Standard deviation, *V5-12 Gy* volumes of healthy brain (brain-gross tumor volume) receiving 5,8,10, and 12 Gy

Median Paddick conformity index was 0.75 for DCAT and 0.60 for VMAT plans (*p* = 0.005); respective median GI indexes were 4.54 (range, 3.45–7.13) and 5.61 (range, 3.64–8.37) (*p* < 0.0001). PTV D_mean_ was 23.3 ± 0.23 Gy for DCAT and 24.0 ± 0.8 Gy for VMAT plans.

**Fig. 1:**
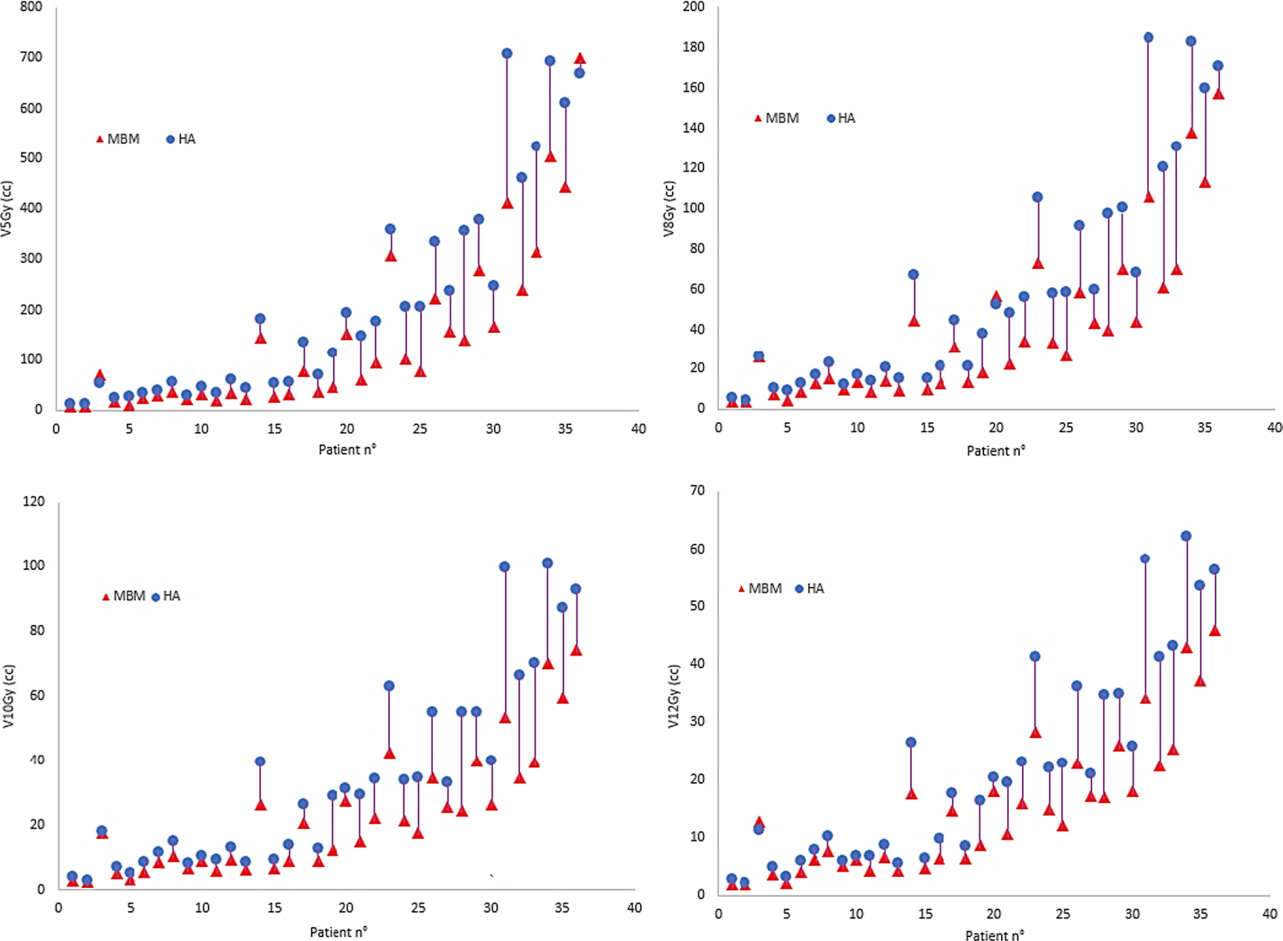
Comparison of 12 Gy, 10 Gy, 8 Gy, and 5 Gy isodose volume for 36 cases between Elements MBM DCAT (▲) and HA VMAT (•) plans

**Fig. 2:**
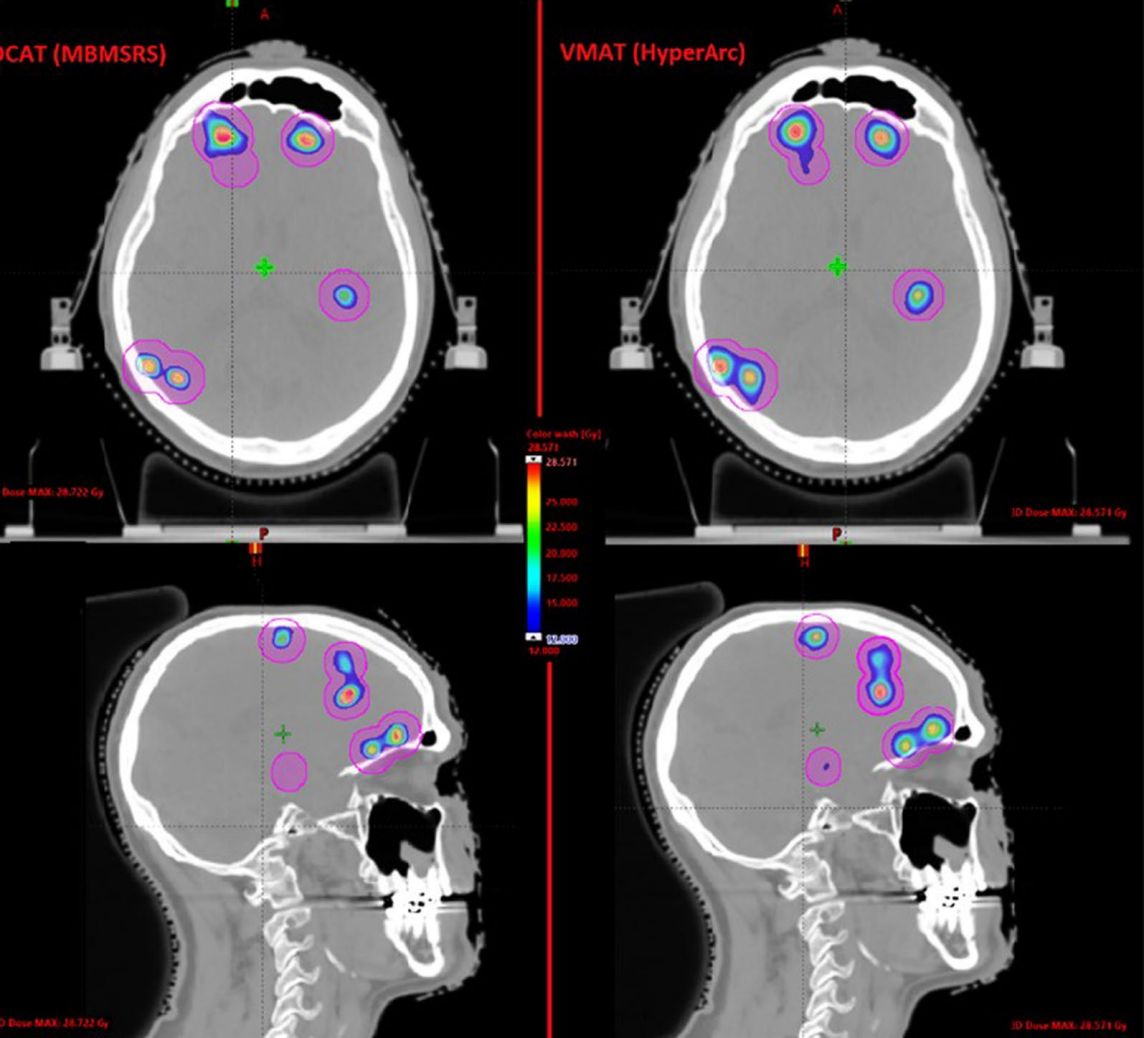
Axial (first row) and sagittal (second row) dose distributions, shown in color wash (12 to 24 Gy), from Elements MBM DCAT (panel A and C) and HA VMAT (panel B and D) plans in a patient (case 36) with 25 brain metastases (only some of them are visible in the selected slices). Total tumor volume was 2.43 cm^3^. A 1-mm GTV-to-PTV margin was used with 20 Gy prescribed to all targets in single fraction. The 12 Gy isodose-shell around the targets with the typical enlargement in case of adjacent lesions, which may bring to dose bridging, is shown for both plans. The volume of normal brain receiving 12 Gy (V_12Gy_) was 45.9 cm^3^ for DCAT plan and 56.3 cm^3^ for VMAT plan. The different management of the dose bridging between the different techniques is noticeable

The median mean dose to the normal brain was 2.32 Gy for DCAT plans and 2.74 Gy for VMAT plans (*p* = 0.0001). Median V_12Gy_ was 12.4 (range, 1.9–45.9) cm^3^ for DCAT plans and 18.5 (2.2–62.3) cm^3^ for VMAT plans (*p* < 0.0001), respectively. Similarly, volumes of healthy brain receiving 10 Gy, 8 Gy, and 5 Gy were significantly lower for DCAT than for VMAT plans (*p* < 0.0001). V_12Gy_, V_10Gy_, V_8Gy_, and V_5Gy_ of individual plans are shown in Fig. 1. When comparing each case, V_12Gy_ was lower for DCAT plans in 35 patients and VMAT plans in one patient, with a median difference between plans of 33%. Median V_12Gy_ differences increased with the number of lesions, 24% for patients with 2–10 lesions and 38% for those with more than 10 lesions. Of note, the largest percent difference > 40% was seen for plans with more than 10 lesions and total tumor volume < 2 cm^3^ (DCAT, 16.8 cm^3^; VMAT, 28.3 cm^3^; *p* = 0.005). Similarly, DCAT plans achieved less V_10Gy,_ V_8Gy_ and V_5Gy_ for 34, 35, and 35 cases; the difference between the mean V_5-12 Gy_ in the two groups was > 30%, with a higher difference in presence of more than 10 lesions (Table 3). An example of DCAT and VMAT plans for a representative patient with 25 brain metastases is shown in Fig. 2.

**Table 3:** Brain dose and V12Gy, V10Gy, V8Gy, and V5 Gy among DCAT and VMAT plans with 1–10 or > 10 lesions

Parameter	DCAT	VMAT	p	DCAT	VMAT	*p*
	1–10 lesions	1–10 lesions		> 10 lesions	> 10 lesions	
*Brain dose*			0.003			0.001
Mean dose (SD)	1.7 (0.6)	1.88 (0.8)		2.64 (1.3)	3.01 (1.5)	
Median dose (SD)	1.63	1.77		2.3	2.7	
Range	0.65–3.0	0.55–3.48		0.65–5.62	0.55–5.85	
*V12Gy*			0.0001			0.001
Mean (SD)	7.7 (5.0)	10.5 (6.8)		25.8 (10.6)	39.7 (13.7)	
Median	6.2	8.2		22.7	38.7	
Range	1.87–18.1	2.2–26.4		12.2–45.9	21.9–62.3	
*V10Gy*			0.0005			0.001
Mean (SD)	10.9 (7.3)	14.7 (10.6)		40.9 (17.5)	63.5 (23.7)	
Median	8.8	11.3		37.4	58.9	
Range	2.6–27.2	2.9–39.6		17.4–74.2	33.7–100.6	
*V8Gy*			0.0001			0.001
Mean (SD)	17.3 (13.5)	25.4 (17.4)		74.0 (40–0)	113.9 (52.8)	
Median	13.2	19.5		62.8	102.7	
Range	3.7–157	4.6–184		27.2–157	58.2–184.0	
*V5Gy*			0.0001			0.001
Mean (SD)	45.7 (40.4)	73.0 (57.6)		291.2 (174.9)	427.8 (183.3)	
Median	31.4	53.9		259.2	368.6	
Range	8.18–153.4	11.3–194.0		77.3–701.8	206.4–707.8	
*Right hippocanpus*			0.004			0.0012
Mean dose (SD)	1.34 (0.8)	1.67 (1.1)		3.8 (1.7)	4.7 (2.1)	
Median dose	1.0	1.54		3.2	3.9	
Range	0.6–3.6	0.6–4.9		1.9–6.9	2.6–8.6	
*Left hippocanpus*			0.03			0.007
Mean dose (SD)	1.69 (0.85)	1.92 (1.2)		3.6 (1.3)	4.7 (1.8)	
Median dose	1.61	1.65		3.0	4.9	
Range	0.5–4.3	0.5–6.2		2.2–6.4	2.99–9.8	

*DCAT* Dynamic Conformal Arc Therapy, *VMAT* Volumetric Modulated Arc Therapy, *GTV* Gross Tumor Volume, *V12Gy*, *V10Gy*, *V8Gy*, and *V5 Gy* volume of normal brain (brain—GTV) receiving 12 Gy, 10 Gy, 8 Gy, and 5 Gy

**Fig. 3:**
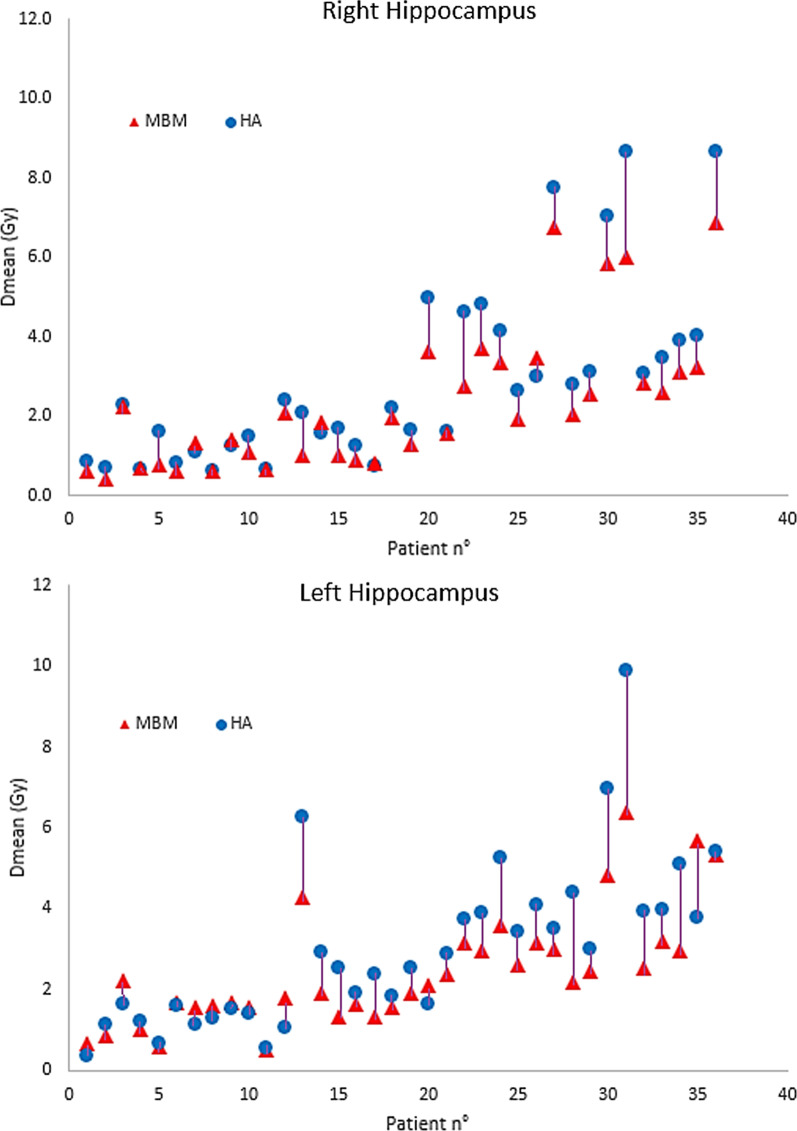
Comparison of mean dose to left and right hippocampus for 36 cases between Elements MBM DCAT (▲) and HA VMAT (•) plans

As shown in Fig. 3, both techniques resulted in excellent sparing of hippocampi, with doses that remained low in plans with either 2–10 or > 10 lesions. Comparative analysis of plans showed significantly lower median doses to either left or right hippocampus for DCAT plans compared to VMAT plans (*p* < 0.0001); for all plans, median difference was around 20% but increased to up 39% in plans with more than 10 lesions. In six patients (No 13, 20, 27, 30, 31, and 36) presenting with at least one lesion in close proximity to the hippocampus (< 5 mm), mean dose to ipsilateral hippocampus was 5.9 ± 1.2 Gy for DCAT and 6.8 ± 1.8 for VMAT plans (*p* = NS). Amongst them, a mean dose > 5 Gy to the bilateral hippocampi was observed in two DCAT and five VMAT plans.

Regarding the indicators of treatment efficiency, DCAT required, for most of patients, 10 arcs and 5 couch yaw rotation angles compared with 5 arcs and 4 yaw rotation angles required for VMAT. Median MUs were lower for VMAT plans compared with DCAT plans (9756 MUs vs 6662 MUs, *p* < 0.0001).

## Discussion

Results of this study, where Elements MBM DCA and Varian Hyperarc VMAT software were compared in patients with 2–25 brain metastases indicate that that high-quality plans can be generated for both treatment planning software, although with a slight superiority of Elements MBM DCAT plans in reducing the volume of irradiated normal brain tissue surrounding the target volumes. V_8-12 Gy_ were significantly lower in DCAT plans compared with VMAT plans, with differences that increased in patients with 10–25 lesions. Such findings may be clinically relevant, because volumes of health brain receiving radiation doses of 10–12 Gy have been associated with a significant risk of radionecrosis after SRS [27, 28]. Following either single-target and multitarget single-isocenter SRS, several studies observed a risk of 5–10% of symptomatic brain necrosis which can significantly increase in patients with V_12Gy_ > 10 ml.

Hofmaier et al. [22] compared Elements MBM plans to Monaco (Elekta, Stockholm, Sweden) VMAT plans in 20 patients with 66 brain metastases. In agreement with our results, the authors observed significant differences in GI and V_10Gy_ and V_12Gy_ between techniques. Elements MBM SRS plans had a lower median GI of 5.99 compared to the GI of 7.17 for the VMAT plans (*p* < 0.05). Median V_10Gy_ and V_12Gy_ were 3.2 and 2.1 cm^3^ and 3.1 and 4.9 cm^3^ in Elements MBM DCAT and Monaco VMAT plans, respectively (*p* < 0.05), being associated with a moderate correlation between the sphericity and differences in V_10Gy_ and V_12Gy_. Similarly, Gevaert et al. [22] reported significantly lower GI, V_10Gy_, and V_12Gy_ for Elements MBM DCAT plans compared to multiple isocenters VMAT plans in 10 patients with up to eight brain metastases.

In contrast, different results have been reported by others [24, 25]. In a study of 20 patients with 2–10 brain metastases, Ruggieri et al. [24] showed no significant differences between Hyperarc and Elements MBM plans for GI, mean dose, although they observed a trend throughout a better V_12Gy_ for Hyperarc VMAT plans, with a decrease in median V_12Gy_ from 37.3 cm^3^ to 23.7 cm^3^ (*p* = 0.06). However, results are hardly comparable with those observed in our and other studies because a significant proportion of lesions in Ruggeri et al. [24] study were treated with 21–27 Gy given in three fractions. In another study comparing the two techniques in 30 patients with up to 10 brain metastases, Liu et al. [25] observed favorable V_12Gy_ for VMAT SRS plans using Varian RapidArc compared with Elements MBM plans (19.2 vs 24 cm^3^; *p* < 0.001). For 24 cases with PTV > 2.1 cm^3^, median V_12Gy_ was significantly lower for VMAT plans, whereas V_12Gy_ favored MBM plans for the remaining plans with PTV < 2.1 cm^3^. These findings conflict with our results. Differences in number and size of lesions may explain, at least in part, the different results. In our series, the largest magnitude of percent difference for V_12Gy_ between DCAT and VMAT plans was seen in patients with 10 or more brain metastases and a total tumor volume < 2 cm^3^, confirming a better performance of DCAT can be observed in patients with low cumulative tumor volume [23, 25].

In our study GTV-to-PTV margin was 1 mm. Using small GTV-to-PTV margins is an essential strategy to reduce the risk of toxicity, since larger margins led to a significant increase of V_12Gy_ [20, 29]. Certainly, the use of 1-mm margins requires robust protocols for quality assurance for all steps of SRS treatment, including the use of contrast-enhanced 3D fast gradient echo T1-weighted sequences with slice thickness of 0.5–1 mm for target delineation, correction of geometric distortion in MR images, and accurate registration of CT and MRI data sets. Precise immobilization and improved patient positioning require sophisticated immobilization and image guidance systems, e.g. orthogonal x-rays (ExacTrac^®^Xray 6D system) cone beam CT (CBCT) [30]. Additional strategy that is expected to reduce the risk of radionecrosis includes the use of fractionated SRS for larger lesions instead of single-fraction SRS.

Other explored dosimetric parameters (isodose metrics) were V_5Gy_ and mean brain doses to assess the low isodose spill. Our results are consistent with those reported in other studies showing that DCAT plans tend to be more favorable for low-dose metrics than VMAT plans [22, 23, 25], with the magnitude of difference for both V_5Gy_ and mean brain doses larger in plans with a higher number of lesions. Similarly, we observed a significantly lower mean hippocampal dose in DCAT plans; however, a mean dose < 3 Gy was achieved in most plans (DCAT,28; VMAT,20), with only two DCAT and five VMAT plans exceeding a mean dose of 5 Gy to the bilateral hippocampi, indicating the robust quality plan for both techniques.

Overall, our data indicate that radiation dose to the healthy brain remains low in patients with up to 25 lesions and this may explain, at least in part, the limited risk of neurocognitive toxicity observed in patients with more than 10 brain metastases who received SIMT SRS, as recently reported [31]. Future studies need to define the relationship between diffuse radiation doses to the normal brain and hippocampi and the development of neurocognitive and quality of life abnormalities following SRS for multiple brain metastases.

Other plan parameters that were evaluated included Paddick CI and GI. The median CI of DCAT plans seen in our study is consistent with the data reported in previous studies [22–25]; however, conflicting results have been reported when comparing the two techniques (Table 4). In 20 patients with 66 brain metastases, Hofmaier et al. [23] observed better conformity for DCAT over VMAT plans; in contrast, a few studies showed significantly better CI for HA VMAT plans over Elements MBM DCAT plans [24, 25]. Conflicting results can be explained, at least in part, by differences in optimization algorithms among treatment planning systems according to the size and shape of lesions, their proximity to OARs, and different centre experience with software optimization. While such indexes remain of interest to assess the dosimetric quality of treatment plans, the impact of such differences on clinical outcomes remains to be demonstrated [32].

**Table 4:** Treatment and dosimetric parameters reported in selected studies comparing VMAT and DCAT SIMT SRS techniques for multiple brain metastases

Parameter	Current study	Gavaert et al. [22]	Hoffmaier et al. [23]	Ruggeri et al. [24]	Liu et al. [25]
No of patients	36	10	20	20	30
No of lesions	367	40	66	97	217
Median number of lesions for patient	9 (2–25)		3 (2–6)	5 (2–10)	7.5 (4–10)
Median SRS (dose/fraction)	20 Gy, SF	20 Gy, SF	19 Gy, SF	18–27 Gy, 1–3 fr	14–24 Gy, SF
Median PTV (cm3)	0.32 (0.17–1.18)	3.15 (0.14–24.61)	0.8 (0.1–11.9)	2 (0.1–18.6)	0.35 (0.014–17.73)
Median total PTV (cm3)	3.42 (0.61–9.37)	10.6 (0.6–28.66)	NR	9.6 (0.5–29)	7.05 (0.49–27.32)
PTV Dmax/D2%	< 135% of PD	< 125% of PD	< 125% of PD	< 150% of PD	< 175% of PD
PTV Dmin/D98%	> 98%	> 99.5%	> 98%	> 98%	NR
Mean target coverage	VMAT 99.1% ± 2.4% DCAT 98.9 ± 2.9%	NR	NR	VMAT 98.2% ± 2.9% DCAT 96.4 ± 3.5%	VMAT 96.9% ± 2.5% DCAT 97.5 ± 2.3%
Median conformity index	VMAT 0.6 (0.46–0.85) DCAT 0.75 (0.52–0.86)	VMAT 0.67 ± 0.16* DCAT 0.65 ± 0.08*	VMAT 0.73 (0.38–0.88) DCAT 0.75 (0.58–0.89)	VMAT 0.94 (0.85–1.00)* DCAT 0.75 (0.4–0.96)*	VMAT 0.77 ± 0.12 DCAT 0.7 ± 0.1
Median gradient index	VMAT 5.61 (3.64–8.37) DCAT 4.54 (3.45–7.13)	VMAT 7.1 ± 3.1* DCAT 3.9 ± 1.4*	VMAT 7.17 (3.35–33.0) DCAT 5.99 (3.5–15.73)	VMAT 4.17 (3.32–7.46)* DCAT 4.31 (3.29–5.79)*	NR
Median brain dose	VMAT 2.74 (0.55 -5.85) DCAT 2.37 (0.65–5.62)	NR	NR	VMAT 2.99 (0.76 -6.17)* DCAT 3.22 (0.85–7.34)*	VMAT 2.76 DCAT 2.57
Median total V12Gy	VMAT 18.51 (2.2–62.3) DCAT 13.65 (1.87–45.9)	VMAT 46.3 ± 35.9* DCAT 36.3 ± 27.1*	VMAT 3.1 (0.5–13.9) DCAT 2.1 (0.1–13.1)	VMAT 23.7 (1.7–86.6)* DCAT 37.3 (3.2–157.5)*	VMAT 19.2 DCAT 23.7

*DCAT* Dynamic Conformal Arc Therapy, *VMAT* Volumetric Target Volume, *SIMT* Single-isocenter multi-targets, *SRS* Stereotactic Radiosurgery, *PTV* Planning Target Volume, *PTV D2*% and *D98*% dose received by 2% and 98% of PTV, *SF* Single Fraction, *V12 Gy* doses of 12 Gy received by healthy brain (brain-gross tumor volume)

*value are reported as mean

In conclusion, both HA and MBM commercially available treatment planning systems were able to generate high-quality mono-isocenter SRS plans in patients with up to twenty-five brain metastases. MBM DCAT SRS treatment plans show superior steeper dose gradients and healthy brain sparing compared with VMAT plans, especially in patients with more than ten lesions and limited total tumor volume. Future studies focusing on optimal patient selection and SRS dose/fractionation should evaluate the clinical impact of mono-isocenter SRS techniques in terms of survival, risk of radiation necrosis and neurocognitive preservation over other approaches.

## Data Availability

All data supporting the results of this review are published in the cited references.
